# Effect of Preharvest Abiotic Stresses on the Accumulation of Bioactive Compounds in Horticultural Produce

**DOI:** 10.3389/fpls.2019.01212

**Published:** 2019-10-04

**Authors:** Stefania Toscano, Alice Trivellini, Giacomo Cocetta, Roberta Bulgari, Alessandra Francini, Daniela Romano, Antonio Ferrante

**Affiliations:** ^1^Department of Agriculture, Food and Environment, Università degli Studi di Catania, Catania, Italy; ^2^Institute of Life Sciences, Scuola Superiore Sant’Anna Pisa, Pisa, Italy; ^3^Department of Agricultural and Environmental Sciences – Production, Landscape, Agroenergy, Università degli Studi di Milano, Milan, Italy

**Keywords:** cold, water stress, light stress, salinity, UV, wounding

## Abstract

The quality of horticultural products is the result of the interaction of different factors, including grower’s crop management ability, genotype, and environment. Sub-optimal environmental conditions during plant growth can induce abiotic stresses and reduce the crop performance with yield reduction and quality losses. However, abiotic stresses can induce several physiological, biochemical, and molecular responses in plants, aiming to cope with the stressful conditions. It is well known that these abiotic stresses are also elicitors of the biosynthesis of many metabolites in plants, including a wide range of bioactive compounds, which firstly serve as functional molecules for crop adaptation, but they have also a great interest for their beneficial effects on human health. Nowadays, the consumer is oriented to low-energy foods with low fat content, but at the same time, growing attention is paid to the presence of bioactive molecules, which are recognized as health-related compounds and concur to the nutraceutical value of plant-derived foods. In this context, fruit and vegetables play an important role as sources of bioactive ingredients in the diet. At the cultivation level, the understanding of crop responses to abiotic stresses and how they act in the biosynthesis/accumulation of these bioactive compounds is crucial. In fact, controlled abiotic stresses can be used as tools for improving the nutraceutical value of fruit and vegetables. This review focuses on the quality of vegetables and fruits as affected by preharvest abiotic stressors, with particular attention to the effect on the nutraceutical aspects.

## Brief Introduction on Abiotic Stress and Crop Responses

Bioactive phytochemical compounds represent non-nutrient plant molecules such as pigments or secondary metabolites (Ismail et al., 2010), influencing the functional and nutritional values of commonly consumed fruit and vegetables commodities due to their established role related to human health and well-being as health-promoting compounds (Liu, 2013).

Abiotic stresses are potent elicitors of bioactive compound biosynthesis, and they should be wisely used for growing crops that are naturally enriched and with high nutraceutical value.

### Plant Metabolism

The main biosynthetic pathway that leads to bioactive molecules accumulation in plants is the phenylpropanoids. These chemical compounds are accumulated in plants with defense or signaling functions. The phenylpropanoids are especially accumulated under stressful conditions, and the different chemical compounds can be associated with specific stresses (Dixon and Paiva, 1995). The shikimate pathway is considered the core of phenylpropanoids biosynthesis. These molecules are classified as secondary metabolites, because they were considered as molecules that do not contribute to the vital processes of plants (Vogt, 2010). The importance of these compounds has been completely revised in plant biology because many molecules of secondary metabolism have a crucial function in plant growth and development such as lignin biosynthesis and its role in plant defense, water, and nutrient transportation. Nevertheless, secondary metabolism is often wrongly considered less important than the primary metabolism. The phenylpropanoids can be absent in different tissues during development, and for this reason, they were considered not essential for plant development. In particular environments, the biosynthesis of phenylpropanoids and related compounds is essential for plant survival. The concentration of phenylpropanoid compounds can vary during plant growth and adaptation to sub-optimal growing conditions. Plants are not able to escape the adverse environmental conditions; therefore, their survival is related to the adaptation ability to different stresses. The plasticity of the plants is associated with the accumulation of bioactive molecules that increase the tolerance to stresses by modulating the main physiological and biochemical processes (Oh et al., 2009a and Oh et al., 2009b). In agricultural systems, the discovery of the traits associated with the crop adaptation strategies can be exploited for reducing yield and quality losses. The tight association of the specific tolerant traits will be used in breeding programs for enhancing the crop performance under abiotic stressful conditions. The yield losses of crops under abiotic stresses have been estimated to be 69% in average (Boyer, 1982; Mariani and Ferrante, 2017).

**Figure 1:**
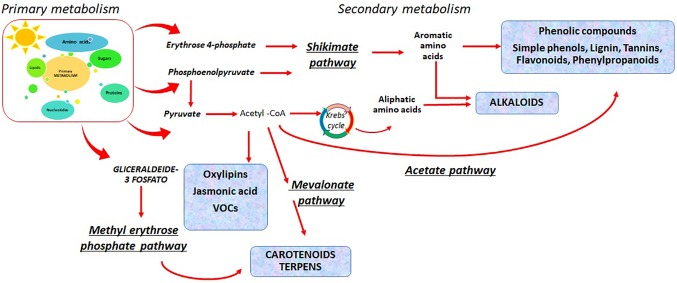
Primary and secondary metabolisms in plants and the different pathways that lead to the bioactive compounds biosynthesis.

Primary metabolism of plants involves photosynthesis and related processes, respiration, sugars (starch and sucrose), and amino acids metabolism (**Figure 1**). Abiotic stresses usually reduce the plant growth by slowing down photosynthesis. Crops invest their energy in defense mechanisms by the activation of specific biosynthetic pathways (Caretto et al., 2015).

Photosynthesis provides the carbohydrates that can supply carbon skeletons for nitrogen assimilation, which leads to amino acids biosynthesis and related bioactive compounds. The amino acids can be used in plants as monomeric molecules for the biosynthesis of proteins or other functional molecules such as nucleic acids, glucosinolates (GSLs), plant hormones, and other nitrogen-containing compounds. Primary metabolism is linked to the secondary metabolism, since several substrates can serve to activate the phenylpropanoids pathway, enabling the biosynthesis of secondary metabolites, including polyphenols. Phenolic compound biosynthesis has erythrose 4-phosphate and phosphoenolpyruvate as first substrates (**Figure 1**). These two metabolites are intermediates of the pentose phosphate pathway and the glycolysis (Vogt, 2010).

The phenylpropanoids biosynthetic pathway is the source of secondary metabolites in plants. These compounds are involved in defense mechanisms and can exert antioxidant functions. Moreover, fruits and vegetables are an important source of these molecules, which in the diet provide beneficial effects on human health.

## The Effect of Salinity on the Nutraceutical Properties of Fruits and Vegetables

High salt concentration in soil and water is a stressful condition that severely affects crop quality and yield. This phenomenon can occur in arid or semi-arid regions as well as in coastal areas, in which the proximity to the sea strongly affects soil and water quality. Moreover, in case of soilless cultivations (such as hydroponically grown leafy greens), the bad quality of the water or the sub-optimal management of the nutrient solutions used can cause a stressful condition for crops. Because of salinity, plants must face a reduction in the water potential of soil and nutritional imbalance, and this turns finally into a decrement in yield and a loss in quality. Plants have developed several strategies to counteract the increment in salt concentration, including the accumulation of osmotically active metabolites, antioxidant compounds, and specific secondary metabolites (Parvaiz and Satyawati, 2008). These strategies aim to reduce the oxidative stress, which could derive from the altered ion and water flux and help to re-establish the water balance within the cell. As a side effect, the changes in the metabolites accumulated in the plant’s edible parts can positively affect the nutraceutical value of the crops, as several stress-related plant metabolites are also appreciated as health-related compounds in human nutrition. For this reason, salt stress, among others, has been recently suggested as a potential eustressor to be used for enhancing the quality of vegetables (Rouphael et al., 2018).

### Salinity Stress and Bioactive Compound Accumulation in Vegetable Crops

Among the leafy vegetables, lettuce is one of the most relevant in terms of economic value and cultivated area; it is also highly appreciated by consumers as fresh-cut commodity. Lettuce is moderately sensitive to salinity; for this reason, the effects of salinity on the nutritional and nutraceutical properties of this crop are widely studied. Salinity affects the mineral composition of leaves and the biosynthesis of health-related phenolic compounds as shown in green and red baby lettuces (Neocleous et al., 2014). An increase in phenols is a common response to salinity in lettuce; in fact, it was also observed in two differently pigmented lettuces subjected to high salinity or CO_2_ levels. In a red-pigmented cultivar, salinity (200 mM) maintained higher phenolic amounts and antioxidant activity. Moreover, in combination with elevated CO_2_ (700 ppm), salinity caused a lower reduction in yield and a higher accumulation of luteolin than does salinity alone (Sgherri et al., 2017). The effect of salinity can vary depending on the species or varieties studies as well as on the salt concentration or the duration of the stress application. For example, the phenolic content of the romaine lettuce (*Lactuca sativa* L. var. *longifolia*) declined as a response to short-term salt irrigation. In the same study, with long-term irrigation with 5 mM of NaCl-enriched water, the total carotenoid (particularly the lutein and β-carotene) content increased without color change (Kim et al., 2008).

Rocket is another leafy vegetable particularly appreciated as fresh-cut salad, rich in phytochemicals such as phenols and GSLs. In wild rocket (*Diplotaxis tenuifolia* L.) plants subjected to a moderate salt stress (200 mM of NaCl for 48 h), the levels of GSLs were reduced, and a key role in the response to salinity has been hypothesized for the gene encoding for the enzyme thio-methyl transferase (Cocetta et al., 2018). Further analyses should be performed to estimate the effect of salinity on the accumulation of GSL-derived products such as isothiocyanates, which have also a proven health-promoting action.

The nutritional quality of broccoli florets was improved under moderate saline stress (40 or 80 mM of NaCl). Salinity caused an increment in phenolic compounds and GSLs, while the mineral composition remained within the range of recommended values (Lopez-Berenguer et al., 2009).

**Figure 2:**
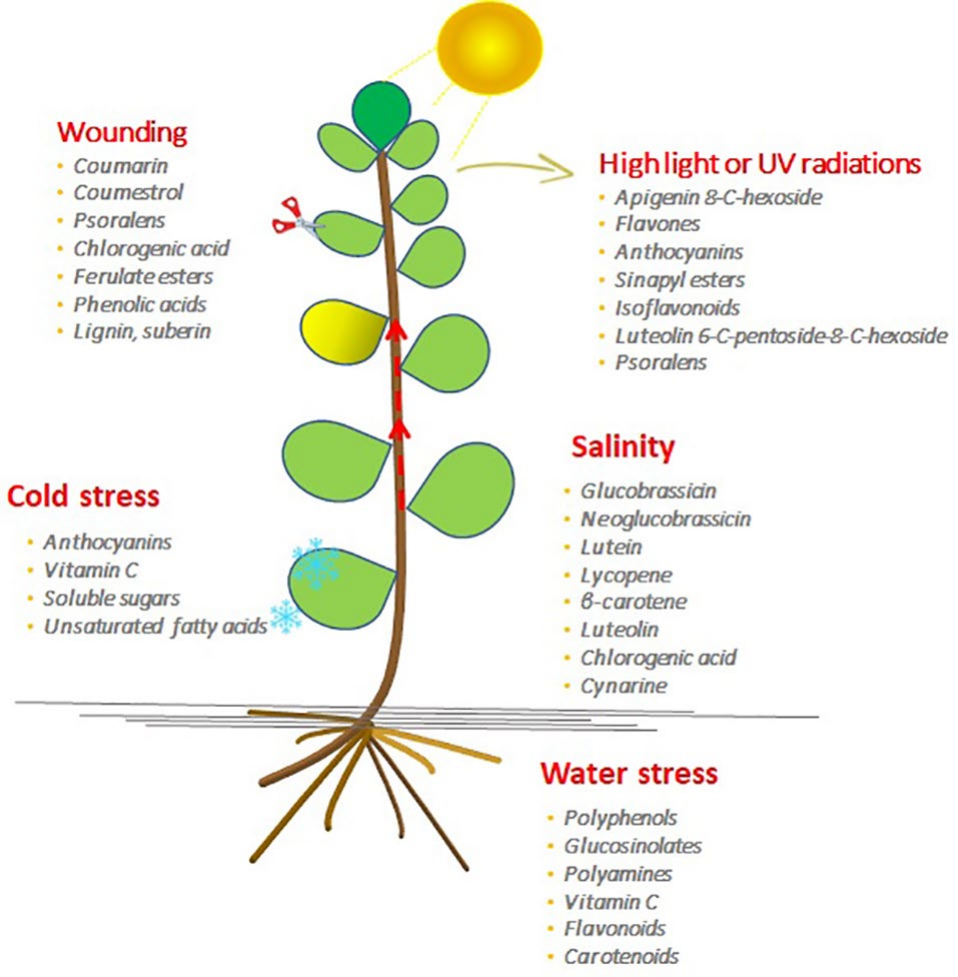
The bioactive molecule accumulation in plants induced by the different abiotic stresses.

The role of certain GSLs in salt stress response in broccoli has been recently shown by Di Gioia et al. (2018). The authors reported that the use of saline water improved broccoli dry matter and soluble solid content, while it had no impact on total GSL concentration. However, salinity induced an increase of indolic GSLs (glucobrassicin and neoglucobrassicin) potentially affecting nutritional properties and flavor (**Figure 2**).

The quality and shelf-life of fresh-cut cauliflowers grown under high salinity was improved; in fact, preharvest salt stress (20 mM of NaCl) increased the concentration of GSLs in a genotype-dependent way, improving also the concentration of total polyphenols and ascorbic acid, hence enhancing the antioxidant activity of florets (Giuffrida et al., 2018).

Colla et al. (2013) studied the effect of increasing salinity (0 and 30 mM of NaCl) applied on artichoke and cultivated cardoon grown in a floating system. Salinity decreased the leaf dry biomass, leaf number, and macroelement and microelement accumulation. On the other hand, antioxidant activity, total polyphenols, chlorogenic acid, cynarine, and luteolin levels were improved in response to salinity.

### Salinity Stress and Bioactive Compound Accumulation in Fruits

Salinity has been reported to affect the nutritional and nutraceutical properties of fruits by inducing metabolic changes in response to the stressful condition. The effect of salinity has been largely studied in tomato fruit and in other important fruit crops (Rouphael et al., 2018). For example, tomato plants grown in a greenhouse were treated with a nutrient solution with electrical conductivities (ECs) of 2, 4, or 6 dS m^−1^, and yield per plant and fruit size were reduced. The antioxidant capacity [oxygen radical absorbance capacity (ORAC)], β-carotene, lycopene, and vitamin C concentrations increased with EC, while lutein was only partially affected by salinity. The same study also showed that salinity does not influence the expression of several key genes involved in antioxidant production in ripe fruit (Ehret et al., 2013). Similarly, Fanasca et al. (2007) showed that the quality of tomato fruits was improved by high EC (2.5 or 8 dS m^−1^ in the root zone). A high EC increased the dry matter content, total soluble solids content, titratable acidity, and glucose, fructose and citric acid contents. Significantly higher lycopene and β-carotene contents were also observed with a high EC.

Salinity stress led in some cases to a two- to three-fold increase in the lycopene content in different tomato genotypes. At the same time, salinity differentially affected the accumulation of total anthocyanins in two anthocyanin-rich tomato genotypes. In fact, anthocyanin content was enhanced in fruits of the genotype ‘Sun Black’ (two-fold increase) and reduced in fruits of ‘Anthocyanin fruit type’ (10-fold decrease) (Borghesi et al., 2011).

Pepper is a good source of nutraceuticals such as ascorbic acid, carotenoids, and phenolics. The effect of salinity depended on the maturity state of peppers, showing a more relevant effect on red fruits. Salinity had no effect on antioxidant activity of the hydrophilic fraction, β-carotene, or sugars, and it decreased ascorbic acid and total phenolic compounds and increased lipophilic antioxidant activity and lycopene (Navarro et al., 2006). A positive effect of salinity on pepper fruit quality was also observed by Giuffrida et al. (2014); the authors showed that total phenol content was slightly increased (+10%) by NaCl salinity and that the concentration of carotenoids was enhanced by 40% to control.

Strawberry fruits obtained from plants subjected to high salinity showed, in some cases, lower total acidity and higher values for soluble solids content; fruit taste was, therefore, enhanced. Moreover, salinity improved the accumulation of antioxidant compounds (Cardeñosa et al., 2015).

Field salinity stress induced an increase of carotenoids and sugars in melon fruits, but at the same time, it has a negative effect in quality and yield, affecting several physiological stress indexes such as malondialdehyde, oxygen peroxide, chlorophylls, and proline (Akrami and Arzani, 2018).

## Water Stress and Produce Quality

Climate change influences changes in rainfall patterns, causing increasing severity of droughts and floods (Sheffield and Wood, 2008). One of the consequences of climate changing is an increase of extreme events, such as drought and waterlogging events. Water stress is one of the most common and dramatic environmental stress in many cultivated areas. It influences plant growth and reduces crop productivity (González-Chavira et al., 2018). The effect of drought stress on plant growth and crop yield depends on the genotype sensitivity, the phenological stage, the organs (leaves and fruits) of the plants, and the intensity and duration of the stress (Marjanović et al., 2012; Mirás-Avalos and Intrigliolo, 2017). Despite the negative effects of water deficit, some studies also reported positive effects on produce quality, such as activating the biosynthesis of secondary metabolites (Sangwan et al., 2001). It has been demonstrated that drought stress can stimulate the metabolism of phytochemicals with health-promoting properties (González-Chavira et al., 2018). Plants stressed by water shortage could represent potential sources of antioxidants such as polyphenols. Consequently, it can be hypothesized that an increase in polyphenols could be obtained with the use of stress-tolerant species (de Abreu and Mazzafera, 2005). The ability of plants to tolerate water scarcity is determined by multiple biochemical mechanisms that improve water retention or uptake, chloroplast functionality, and cell ion homeostasis. One of the main adaptation strategies is the biosynthesis of osmotically active molecules that control the flow of ions and water, eliminating oxygen radicals that can function as chaperones (Munns, 2002; Kapoor et al., 2015). Beyond the type of vegetables (leaf or fruit), the increase of bioactive compounds is connected to the reduction of weight increase of the different organs, for which the percentage incidence of phytochemicals increases, but not the content total of bioactive compounds. In any case, the differences found, beyond the level of water stress imposed, are a function of the characteristics of the organs used and of the various vegetable species.

### Bioactive Compounds in Leafy Vegetable Quality

Among the leafy vegetables, particular attention has been paid to Brassicaceae vegetables for the relevance of their health products. The cultivation under stress conditions can stimulate the biosynthesis of bioactive compounds (Oh et al., 2009b), often associated with antioxidant systems linked to plant defense mechanisms (Mittler, 2002).

In broccoli, the most effective abiotic stress that can affect the content of bioactive phytochemicals is not yet clear. This could be related to the fact that stress affects specific phenolic compounds (i.e., phenolic acids), although the magnitude of the effect depends on the cultivar and the plant organ considered (Domínguez-Perles et al., 2010). Thus, Fortier et al. (2010) observed a higher content of phenolic compounds in broccoli subjected to water and nitrogen stress (Khan et al., 2011). The increase in the phenolic content could be an indirect effect of drought stress, due to the higher temperatures of plants under reduced evapotranspiration (Krumbein et al., 2007). An increase of phenolic content was observed in rapeseed (*Brassica napus* L.) during flowering and pod fill under drought stress (Jeffery et al., 2003). In broccoli, besides phenolic compounds, water deficit also induced anthocyanin accumulation (Chalker-Scott, 1999; Rodríguez-Hernández et al., 2012).

In a study focused on the possible role of six different *Brassica* vegetables (*Brassica oleracea* L. and *Brassica rapa* L.) as a natural source of antioxidant compounds, an increase in total flavonoids and l-ascorbic acid was found few days before harvest when plants were subjected to high water deficit (Aires et al., 2011). An increase in vitamin C content with moderate water deficits was found in broccoli (Toivonen et al., 1994) and leeks (*Allium porrum* L.) (Ahmed et al., 2014), which led to the hypothesis that a high content of vitamin C acts as a protective strategy against drought damage (Bozhenko, 1965).

On the contrary, also, the excess of water can induce the activation of the biosynthesis of bioactive molecules. In onion (*Allium cepa* L.), waterlogging reduced bulb quality traits such as phenol, pyruvic acid, flavonoids, antioxidant activity, and total soluble solids content (Ghodke et al., 2018).

In broccoli during drought conditions, higher kaempferol levels in well-watered plants were found, while stressed plants showed a decreased biomass production. The higher kaempferol content in drought and water-logged plants suggests that plants produced kaempferol as a biochemical adaptation towards water stress (**Figure 2**). Decreased plant growth might be related to higher kaempferol content, as plants need to invest photosynthates as resources of carbon required for the kaempferol biosynthesis; defensive flavonoids are expensive for the plants, and their accumulation causes a reduction of the plant’s growth (Gayler et al., 2004). Robbins et al. (2005) observed that water stress decreased GSL content relative to unstressed broccoli (Pék et al., 2012). In broccoli, a higher level of water supply determined an increase of GSL level, making plants more tolerant of pest and insects (Khan et al., 2011).

Schreiner et al. (2009) analyzed the water stress response of two lines of Ethiopian mustard (*Brassica carinata*), in order to evaluate the GSL metabolism response (**Figure 2**). In both lines, 2-propenyl glucosinolate and 3-indolyl methyl glucosinolate were the most representative GSLs with an increase of 80% and 120%, respectively, in both lines. The increase was inversely correlated to the soil water content with severe yield losses (Nora et al., 2012).

An accumulation of GSLs in rapeseed leaves (*B. napus*), grown in low water potential, was found (Jensen et al., 1996). Unfortunately, it is not always possible to distinguish whether increases in GSL concentrations are related to a real increase in GSL biosynthesis or are due to lower biomass production, while the quantity of GSLs produced is not affected (Bloem et al., 2014). However, the answer is complex and depends on the plant phenophase. If the water stress, in fact, was determined in the branching phase, the concentration of glucotropaeoline (GT) was higher, while in the subsequent phenophases, the increases were smaller (Bloem et al., 2014). In rapeseed, there was a 50% increase in GSLs if water unavailability occurred after flowering (Mailer and Cornish, 1987). This could explain, at least in part, the contradictory conclusions published. While Robbins et al. (2005) found a reduction in the concentration of GSLs in drought-stressed broccoli, Schreiner et al. (2009) found an increase in GSL concentration in *B. carinata* with a soil water content of less than 80%, and Radovich et al. (2005) found the increase in GSL in cabbage (*B. oleracea* L. Capitata Group).

In *B. napus*, waterlogging affected the oil quality by increasing erucic acid (C22:1) and GSL content. Waterlogging also caused an increase in linolenic acid (C18:3) and a decrease in linoleic acid (C18:2), indicating that this kind of stress might affect metabolic pathways involving lipid biosynthesis (Xu et al., 2015).

In nasturtium (*Tropaeolum majus* L.), moderate drought stress (65–70% of the amount of irrigation water that was applied to the control plants) and the application of methyl jasmonate (MeJA) were considered as suitable tools to increase the GT content, representing the sole GSL measured as target compound (Bloem et al., 2014).

In lettuce (*Lactuca sativa* L.), interesting bioactive compounds for the human diet are, among others phenolic substances, carotenoids and dietary fiber (Schreiner and Huyskens-Keil, 2006). Oh et al. (2010) found increasing levels of phenolics during drought stress in lettuce. In American lettuce, grown in a greenhouse and subjected to four levels (25%, 50%, 75%, and 100%) of evaporation restitution, it was noted that the content of each amine, with the exception of agmatine, increased with stress water (Coelho et al., 2005). Polyamines can eliminate free radicals, protecting the membranes from lipid peroxidation and oxidative stresses. Lettuce contains two main classes of phenols and polyphenols: caffeic acid derivatives (Ke and Saltveit, 1988) and flavonols (Hermann, 1976). The amount of these antioxidants and micronutrients in the leaves is modified by the cultivar (Llorach et al., 2008), growth conditions, and environmental stresses (Galieni et al., 2015).

In two cultivars of green leaf lettuce (‘Lollo Bionda’ and ‘Vera’), subjected to different levels of water deficit (25%, 50%, and 75% management allowable depletion [MAD] levels), it was noted that 50% of MAD caused an increase in chicoric acid, caftaric acid, chlorogenic acid, and caffeic acid, while 75% of MAD increased levels of kaempferol, quercetin, and myricetin (Malejane et al., 2018). The content of ascorbic acid, on the other hand, decreased with increasing levels of MAD. The water deficit, therefore, stimulates the biosynthesis of phytochemicals in plants and improves the quality of crops. Water stress also induces gene expression and the enzymatic activity of phenylalanine ammonia lyase (PAL; E.C. 4.3.1.5), the primary enzyme of the phenylpropanoid pathway (**Figure 1**), responsible for the biosynthesis of phenolic compounds (Oh et al., 2010). In this study, also the antioxidant capacity [ferric reducing antioxidant power (FRAP) method] appeared higher with MAD at 50%, probably due to the increase in hydroxycinnamates (Malejane et al., 2018). Among the lettuce cultivars, ‘Vera’ was the most suitable cultivar for deficit irrigation (DI), due to its increase in phytochemicals and the quality of the crop without compromising the fresh biomass. In iceberg lettuce, DI has led to a reduction in chlorogenic acid and an increase in chicoric acid (Luna et al., 2012). In contrast, Oh et al. (2010) found, also in lettuce, an increase in total phenolic concentration and antioxidant capacity in the presence of water stress.

Sucrose, glucose, and fructose are soluble sugars in plants. The first two sugars participate as substrates for cellular respiration or as osmolytes to maintain cell homeostasis (Gupta and Kaur, 2005). An increase in fructose, instead, is connected to the phenolic compound biosynthesis (Hilal et al., 2004) rather than provides osmoprotection (Rosa et al., 2009). Like drought, oxidative stress in photosynthetic tissue might result in the decomposition of carotenoids, which was found in lettuce in the water-logged treatment (Eichholz et al., 2014). The phenol content, in particular the chicoric acid, is increased under water stress (Oh et al., 2010).

‘Teodore’ lettuce showed significantly lower contents of β-carotene under waterlogged conditions compared with the well-watered treatment (−27%). Under waterlogged conditions, neither phenolic compounds nor dietary fiber were influenced in their contents (Eichholz et al., 2014).

Secondary compounds, essential oils, and aromatic components of leaves often increase due to environmental stress. An increase in antioxidant compounds was observed in Oman basil plants (*Ocimum basilicum*) subjected to various water regimens [from 65 (12.5%) to 500 ml/day (100%)]. The maximum amount of total phenols and total flavonoids was observed with an irrigation intensity of 25%. Also, the DPPH scavenging activity and the reduction of the antioxidant capacity of the basil leaf extract were also higher with 25% water regimen. A further reduction in the availability of water up to 12% has instead caused a reduction in antioxidant compounds and antioxidant activities (Khan et al., 2012).

Short-term exposure to moderate water stress in thyme (*Thymus vulgaris*), nipplewort (*Chelidonium majus*), and parsley (*Petroselinum crispum*) increased concentrations and overall content of the related secondary metabolites in *T. vulgaris* and *C. majus*. However, longer periods of drought have led to a clear reduction in the overall content of the metabolites also due to greater growth reductions. *P. crispum*, very sensitive to drought, even with short-term stress, has experienced significant reductions in growth (Kleinwächter et al., 2015).

### Bioactive Compounds in Fruit Vegetable Quality

Tomato (*Solanum lycopersicum* L.) is a crop in which the relationship between phytochemical production and water stress has been deeply analyzed for the relevance of health effects of its fruit and the interest of cultivation with reduced quantity of water (i.e., DI).

DI, in fact, can be used both to save water and also to improve the quality of some products; nevertheless, special attention must be given when stress was induced, because the water deficit in sensitive phenological phases (such as flowering) can reduce the content of sugars, acids, and carotenoids (Ripoll et al., 2016). Different authors observed how DI increased the content of lycopene, vitamin C, and β-carotene (Favati et al., 2009; Patanè and Cosentino, 2010; Patanè et al., 2011; Chen et al., 2013), regardless of dependence on period and the degree of water stress (Pulupol et al., 1996; Nuruddin et al., 2003; Marouelli et al., 2004; Favati et al., 2009). DI could, therefore, be considered a useful tool to increase in tomato fruits the content of these nutrients (Juroszek et al., 2009), although it reduces the yield (Nuruddin et al., 2003; Marouelli et al., 2004).

Regulated DI (RDI) increased the content of several secondary metabolites (carotenoids and phenolics) in different cultivars of tomato (‘Tigerella’, ‘Palamós’, ‘Byelsa’, ‘Lazarino’, and ‘Summerbrix’). The response depended on the cultivar and linked to cultivar resistance to water deficit; for example, ‘Palamós’ did not change total carotenoid, while ‘Summerbrix’, ‘Tigerella’, and ‘Palamós’ did not modify the total phenolic compounds (Coyago-Cruz et al., 2017).

Riso et al. (2004) observed that the phytochemicals in tomatoes, in particular lycopene, reduces reactive oxygen species (ROS), thus avoiding cellular damage; however, several other mechanisms of healthy carotenoid action have been suggested (Krinsky and Johnson, 2005). With lower water availability, a higher ascorbic acid content was found during fruit ripening (Kumar et al., 2012). In a study conducted by Helyes et al. (2014), the β-carotene level was positively influenced by water stress. In particular, in the first ripening stages, β-carotene/lycopene ratio was also influenced by drought stress, and results suggested that this stress preferably induces the carotenoid biosynthetic pathway of β-carotene (Riggi et al., 2008). Also, Favati et al. (2009) found that the lycopene and β-carotene levels were higher during drought stress conditions.

Four tomato cultivars were grown in a greenhouse under drought conditions compared with well-watered conditions. At the end of the experiment, the drought stress caused significant differences in antioxidant compositions (lycopene, total phenolics, and flavonoids) and antioxidant activities (DPPH and ABTS) (Klunklin and Savage, 2017).

Drought stress, imposed on five tomato cultivars (‘Kosaco’, ‘Josefina’, ‘Katalina’, ‘Salomé’, and ‘Zarina’), caused an increase in phenolic compounds, especially in flavonoids, only in ‘Zarina’. This increase was correlated to DAHPS activity (+33%) (Sánchez-Rodríguez et al. (2011).

Phenolic compounds (phenolic acids and derivatives) and polyphenols (flavonoids and polymeric compounds) play an important role in detoxification of free radicals (Ksouri et al., 2007). Water stress, like numerous other environmental stresses, which determine the accumulation of ROS in plants, can increase these scavenger molecules.

The activity of the key enzymes of the phenylpropanoid pathway also intensifies in response to environmental stresses (Weisshaar and Jenkins, 1998), whereby PAL has been associated with greater resistance to drought stress (Keleş and Oncel, 2002), as was demonstrated by the response of PAL-deficient mutants (Gitz III et al., 2004).

Water-deficit conditions generally determine the production of fruits with a higher antioxidant activity, due to a decrease in enzymatic activity and an increase in vitamin C and total phenolic content (Helyes et al., 2012; Barbagallo et al., 2013; Pék et al., 2014).

In tomato, waterlogging reduced the total sugar content (Singh et al., 2017).

Hot pepper subjected to deficit of water (100%, 85%, 70%, and 55% of water-holding capacity) showed a reduction of vitamin C content in DI treatments (Ahmed et al., 2014). Other studies (Mahendran and Bandara, 2000) on chili cultivars demonstrated the negative effect of drought stress on vitamin C content. Marín et al. (2009), indeed, have showed an increase of 23% of this vitamin in peppers subjected to water deficit.

Sweet peppers (*Capsicum annuum*) subjected to drought stress increased the phytochemical contents (phenols and anthocyanins) (Dixon and Paiva, 1995). In this sense, as reported by Sánchez-Rodríguez et al. (2012), water stress could represent a suitable tool for managing plant growth and enhancing fruit quality. In fact, López-Marín et al. (2017) showed that the water deficit generally increases the chemical parameters of product quality. As previously reported, the stress response is cultivar dependent and is related to the harvest stage. In a study conducted by Marín et al. (2009), green peppers showed an increased in vitamin C content by 23%, whereas red fruits showed an increase in total carotenoids and provitamin A (30% and 15% respectively) as a consequence of DI. Furthermore, an increase of 30% in total carotenoids was observed in pepper red fruits subjected to water stress.

In pepper, waterlogging significantly reduced soluble proteins, soluble sugars, free amino acids, P, Fe, vitamin C, and vitamin E contents of the fruit (Ou et al., 2017).

Since the strawberry is sensitive to water scarcity, especially during flowering and ripening phase, cultivation is carried out under irrigated conditions (Krüger et al., 1999). Strawberry fruits treated by DI had increased concentration of some compounds linked to taste and health. In strawberry subjected to DI, higher concentrations of anthocyanins and antioxidant (Terry et al., 2007; Giné-Bordonaba and Terry, 2016) and ascorbic acid (Bordonaba and Terry, 2010) were found.

Watermelon is a natural source of lycopene, vitamin C, and l-citrulline (Fish and Bruton, 2014). In drought stress conditions, l-citrulline protects plants from oxidative stress (Akashi et al., 2001; Yokota et al., 2002). In fact, during drought stress, there is an accumulation of l-citrulline, which limits oxidative stress (Akashi et al., 2001) and the development of osmotic pressure and reduces the mechanical characteristics of the pulp (Soteriou and Kyriacou, 2015). Full irrigation treatment in watermelon reduced the vitamin C content, while the different irrigation treatments did not influence the lycopene content (Kuşçu et al., 2015); Leskovar et al. (2004), instead, found that watermelon cultivated in DI showed an increase of quality characteristics (lycopene and vitamin C content).

Drought stress induced an increase of total soluble solids content (23%) and β-carotene content (25%) in ‘Mission’ (muskmelon; reticulatus) and ‘Da Vinci’ (Tuscan; reticulatus), respectively (Sharma et al., 2014).

In a study by Balakumar et al. (1993), drought stress in cowpea plants (*Vigna unguiculata*) increased anthocyanin levels. Drought stress involves the water migration from cells, causing dehydration and plasmolysis. Given the induction from osmotic stress, it is not surprising to find that plants that are resistant to drought stress contain elevated contents in anthocyanins (Chalker-Scott, 1999).

## Cold and Heat Stress Effects on Produce Quality

### Cold Stress and Accumulation of Bioactive Compounds in Crops

The exposure to low but non-freezing temperatures, called cold stress (Raison and Lyons, 1986), can cause severe crop losses. Among the numerous phenotypic symptoms observable after cold stress exposure, we can list poor seed germination, stunted seedlings, leaf senescence, reduction in the leaf expansion, increase of wilted leaves, and finally the death of tissues. Severe membrane damage may also occur in case of cold stress (Galindo et al., 2007; Yadav, 2010). However, moderate stress conditions could induce in plants the accumulation of antioxidants and secondary metabolites as a defense mechanism (Rivero et al., 2001). The use of controlled abiotic stresses may represent an alternative strategy to increase the presence of healthy plant compounds in many vegetables and fruits (Rajashekar et al., 2009), and this issue is of interest to both producers and consumers. In fact, various crops exposed to cold stress have been shown to have higher nutritional values (Yoon et al., 2017, and references therein). Usually, in conditions of low growth temperatures, plants tend to increase the concentration of soluble sugars to promote osmotic adjustment, enhancing freezing tolerance. These findings are reported in a work conducted on spinach (*Spinacia oleracea* L.), cultivated in greenhouse and exposed to cold stress (Yoon et al., 2017). For humans, sugars are compounds with nutritional value, and they provide energy to the human body. The accumulation of soluble sugars could also affect taste, by increasing sweetness, and leaf tenderness (Decoteau, 2000; Watanabe and Ayugase, 2015). In kale leaves, it was observed that low temperatures increased soluble sugar content (Hagen et al., 2009), and an increment in the content of health-promoting phenolic compounds, in particular flavonols, was also reported (Neugart et al., 2012). Ascorbic acid accumulation is favored at lower temperatures. Cold stress exposure enhanced by almost double the vitamin C levels in spinach leaves cultivated in greenhouse as observed by Yoon et al. (2017); also, Watanabe and Ayugase (2015) found that the nutritional quality of winter sweet spinach (*S. oleracea* L.) was higher if the crop was subjected to low temperatures, considering the abundance in ascorbic acid resulting from chilling stress. Proietti et al. (2009) observed that the exposure at 10°C increased the total ascorbic acid concentration in spinach leaves by 41% compared with the levels registered in plants grown at 25°C/20°C day/night temperature regimen. Pak Choi plants (*Brassica rapa* ssp. *chinensis* L.) grown in greenhouse with three mean temperature settings (18°C, 21°C, and 25°C) showed increased ascorbic acid content at sub-optimal growing temperature exposure (Mahmud et al., 1999). As reported by Oh et al. (2009a), the total phenolic content increased in response to chilling (4°C for 1 day) in lettuce plants (*Lactuca sativa* L.) grown in a growth chamber. A significant increment was observed within 1 h of chilling, and the highest phenolic compound level occurred after 3 days of plant recovery. Similarly, in lettuce plants, the antioxidant capacity showed an increment when stress conditions occurred. In three cultivars of Japanese parsley (*Oenanthe stolonifera* D.C.) (Hasegawa et al., 2001) and in strawberry cell suspension cultures (Zhang et al., 1997), it was found that low temperatures caused an accumulation of anthocyanins. Phenolic compounds are important for the appearance, taste, flavor, and aroma of food products, as well as for their health-promoting aspects (Tomás-Barberán and Espín, 2001).

Watermelon plants (*Citrullus lanatus* [Thomb.] Mansf. cv. Dulce Maravilla), in a condition of sub-optimal growth temperature (15°C), showed increased levels of phenolic compounds, as an adaptation strategy to cold stress. At this temperature, also, the highest PAL activity was registered (Rivero et al., 2001). Also, changes in the composition of membrane fatty acids are plants’ strategy to enhance membrane stability, integrity, and function by increasing the proportion of the unsaturated fatty acids. These cold-enhanced polyunsaturated fatty acids and their higher intake are also linked to several health-promoting benefits for humans, in particular related to a reduced risk of both cardiovascular diseases and cancers (De Lorgeril and Salen, 2012).

In apple fruits, the low temperatures or high carbon dioxide during storage induced the accumulation of 4-aminobutyrate and 4-hydroxybutyrate (Brikis et al., 2018). These metabolites seem to be correlated to multiple abiotic stresses and play an important role in the regulation of transcriptional and biochemical mechanisms, which lead to the accumulation of bioactive compounds.

Low temperatures can also activate specific genes that can enhance the tolerance of plants to the freezing temperature. In grapevine, it has been observed that in chilling temperature compared with freezing conditions at the transcriptomic level, the plants showed different patterns of transcript profiles and enriched pathway responses relative to bioactive compounds. The most differentially expressed genes were those that belonged to the ethylene signaling; ABA signaling; the AP2/ERF, WRKY, and NAC transcription factor families; and the sugar pathways (Londo et al., 2018).

### Heat Stress and Bioactive Compounds in Crops

Heat stress is a common abiotic stress in Mediterranean areas and in crops grown in greenhouse or plastic tunnels during spring–summer. High temperatures directly affect plant metabolism, acting on the enzyme activities. Therefore, many physiological processes are slowed down or impaired. Photosynthesis and phenylpropanoid pathways are primarily affected by heat stress. In particular, high temperatures can induce the accumulation of antioxidant in order to protect the cell membrane from breakdown and peroxidation. Heat stress usually induces the accumulation of ROS and the activation of detoxification systems. Tomato plants exposed to 35°C showed an increase of ascorbic acid (vitamin C) and improved the activity of the ascorbate/glutathione-related enzymes (Rivero et al., 2004). Heat-stressed plants undergo changes in carbon metabolism including the increase of soluble sugars. The stressed plants show also an increase in bioactive compounds such as proline, glycine betaine, and sugar alcohols (Wahid et al., 2007). The main function of these molecules is to stabilize proteins/enzymes and the membrane bilayer structure of plant tissues under heat stress conditions.

The phenolic concentrations in heat-stressed plants can have different behaviors depending on the species and their tolerance or sensitivity to high temperatures. Tomato plants exposed to 35°C showed a significant increase of total phenols, while watermelon showed a reduction of these compounds. In both species, the increase or decrease of total phenols was correlated to the higher or lower PAL enzyme activity (Sánchez-Rodríguez et al., 2010), confirming a key role for this enzyme in regulating plant stress responses.

## Light Sub-Optimal Stress and Produce Quality

The light is the most important energy source for plant growth, influencing morphogenesis and yield, and its signaling pathway plays a key role in the modulation of phytochemical profile through a light-mediated metabolic reprogramming (Ghasemzadeh et al., 2010; Karimi et al., 2013; Bian et al., 2015). In horticulture products, the bioactive phytochemical profile is directly or indirectly influenced by light intensity, which can be either a limiting or promoting factor for bioactive compounds accumulation (**Figure 2**).

**Figure 3:**
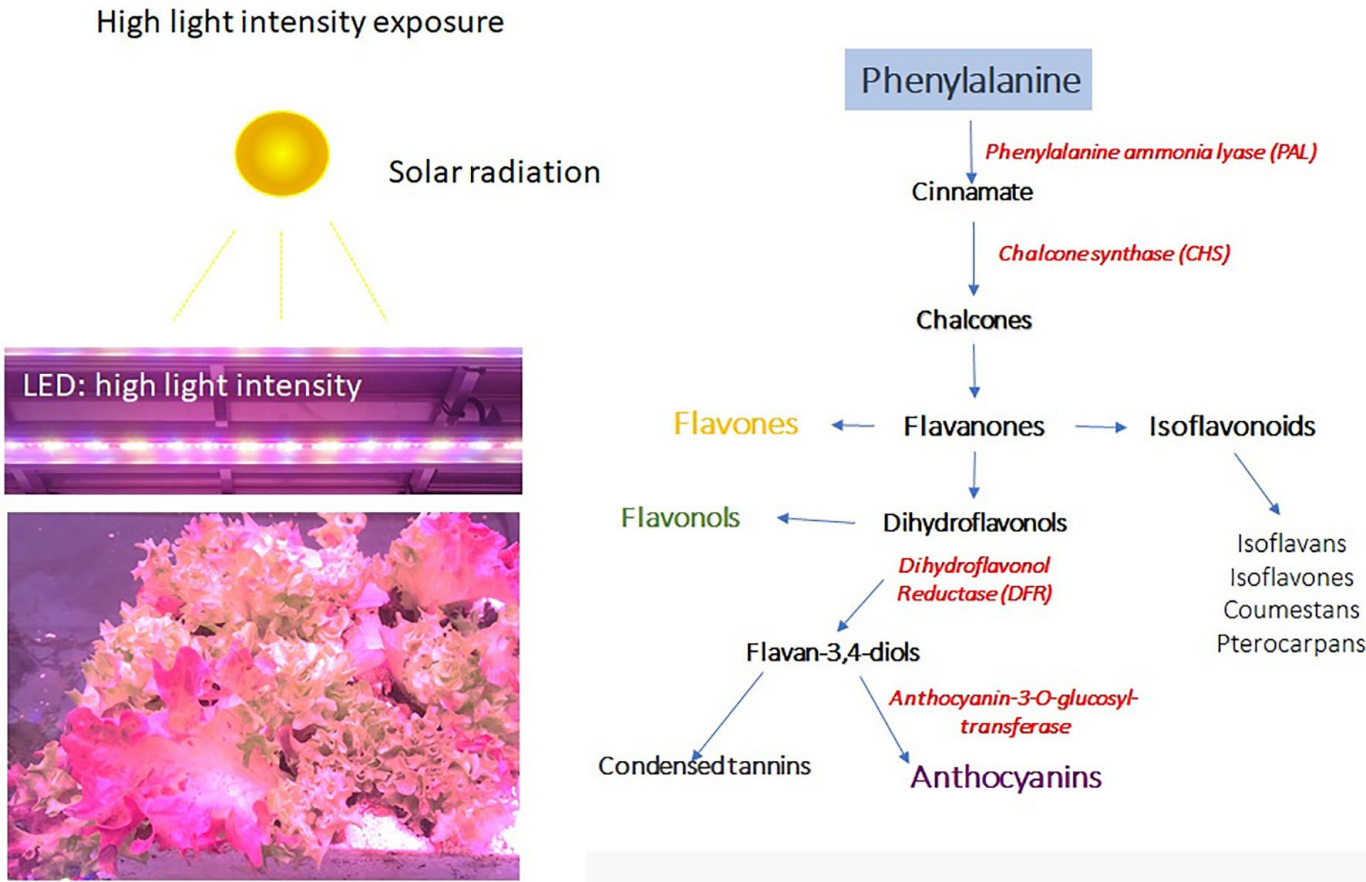
High solar radiation or artificial lighting can induce the biosynthesis of anthocyanins and other phenylpropanoid compounds.

Generally, the light excess can induce severe damage to the photosynthetic apparatus and can compromise the quality of plants, but it depends on the time and intensity. A prolonged exposure of plants to excessive radiation results in light-induced inactivation of photosystem II (PSII), which may cause the death of the organism when the rate of damage exceeds the rate of repair process, leading to complete disintegration of chlorophyll protein complexes (Kataria et al., 2014). The partial or complete photo-destruction of these pigments (chlorophyll photo-bleaching) are strictly related to a reduction of carotenoids content because of their failure to provide, in this condition, protection from photooxidative degradation (Kataria et al., 2014). Under this condition, the photosynthesis is inhibited (Jordan et al., 1994; Sztatelman et al., 2015), resulting in an increase in ROS production and finally death of the photosynthetic organism (Apel and Hirt, 2004). However, plants develop different photoprotection strategies to dissipate the excess of light energy when exposed to high light levels favorable to photoinhibition (i.e., light absorbed exceeds the capacity of photosynthesis), for example, through the xanthophyll cycle. The constituents of photosynthetic protein pigment LHCII complexes (the xanthophyll pool) increase as a photoprotective mechanism to prevent the light-induced damage of the photosynthetic machinery due to the formation of ROS under excess light (Yoo et al., 2003), resulting in an increment of carotenoids to reinforce preservation against photodamage (Yoo et al., 2003; Fanciullino et al., 2014). Generally, along with antioxidant activity of carotenoids, other lipophilic antioxidants are involved as showed in pea (*Pisum sativum*) grown under high irradiance (Ariz et al., 2010) as well as in spinach and lettuce (Oyama et al., 1999). Nonetheless, light exposure appears to have little or no effect on carotenoid content of the majority of the edible portion of fruits and vegetables (Kalt, 2005), whereas activation of secondary metabolism, and in particular, the phenylpropanoid pathway (**Figure 3**), can induce the biosynthesis of a wide range of bioactive compounds by exposure to elevated light in horticultural products. Polyphenols contribute to the increase in quality of fruit, vegetable, and their processed products (Heim et al., 2002; Cheynier et al., 2006); and it is widely accepted that high light intensity acts as stress inducer driving their biosynthesis (Ramakrishna and Ravishankar, 2011). These bioactive compounds in fact may function as a scavenger and as a protective screen layer against harmful high-energy radiation (Agati and Tattini, 2010).

In lettuce plants, the exposure to mild to high light (800 μmol m^2^ s^−1^ for 1 day) stresses induced the highest accumulation of all the phenolic compounds examined (caffeic acid derivatives, chicoric acid, and chlorogenic acid), showing a phenolic compound level six-fold higher in treated plants than in the control plants (Oh et al., 2009a). The influence of fertilization, light, and temperature on flavonoids and caffeoyl derivatives and related gene expression was investigated in tomato (*Solanum lycopersicum*, ‘Suzanne’). In this study, PAL activity, and expression levels of chalcone synthase (CHS2) and flavanone 3-hydroxylase (F3H) genes were significantly enhanced at a higher light intensity, in agreement with a corresponding increase in flavonoid and caffeoyl content (Løvdal et al., 2010). Grape berry (*Vitis vinifera* L.) development and its phenolic compound accumulation are influenced by light environment and are considered to have a significant impact on wine quality (Downey et al., 2006). The light-induced phenolic metabolism and transcriptome changes were recently analyzed in Cabernet Sauvignon grape berries under sunlight exposure treatments at different phenological stages (Sun et al., 2017). In this study, the accumulation and compositional changes of hydroxycinnamic acids and flavonoids drastically increased by sunlight in exposed grape berries, and this metabolic reprogramming correlated well with transcriptional network changes of genes coding PAL, 4-coumarate:CoA ligase (4CL), flavanone 3-hydroxylase (F3H), and flavonol synthase (FLS) family members and their regulatory TF genes, highlighting the importance of theoretical foundations for cultural practice and environmental impact in wine production.

On the other hand, low light availability is the limiting factor for plant production due to adverse environmental conditions (cloudy days) or light-block horticulture facilities (greenhouses). Physiological and morphological traits such as photosynthesis, carbon and nitrogen fixation, leaf morphology and anatomy, gas exchange, and water relations (water use efficiency, stomatal conductance, and thus photosynthesis) are substantially affected in horticulture crops by ambient low light stress (Bjorkman, 1981; Atanasova et al., 2003; Heuvel et al., 2004; Gregoriu et al., 2007). Thus, horticulture products that were grown under low irradiance showed a decrease in flowering, fruit set, and fruit size (Passam and Khah, 1992; Pearson, 1992; Hampson et al., 1996).

In addition, low light intensities determine a metabolic reprogramming, leading an adjustment in the antioxidant profiles and pigment contents (Gruda, 2005). Vitamin C, including ascorbic acid and dehydroascorbic acid, is one of the most important and powerful antioxidants provided by fruits and vegetables having many biological activities in the human body (Lee and Kader, 2000). It is well known that the lower the light intensity, the lower the content of ascorbic acid in many fruits and vegetables (Weston and Barth, 1997; Lee and Kader, 2000). Leaves of lettuce grown with increasing levels of shading manifested a narrow shape and a reduced fresh weight and leaf number, and their sugar and ascorbic acid contents significantly decreased (Shinohara and Suzuki,1981). A similar relationship between the light conditions and the ascorbic acid content was observed for different fruit and green leafy vegetables (Shinohara, 1987; Oyama et al., 1999; Weerakkody, 2003). For example, harvested strawberry fruits in spring showed a higher content of ascorbic acid together with other nutrients than did those harvested in autumn (Caruso et al., 2003), highlighting a seasonal influence on the amount of vitamin C formed.

The color of fruits and vegetables is a key and critical factor to consumer acceptance and the marketing success of these products, and it is dependent on light irradiation intensity (Gironi and Testoni, 1990; Kays, 1999; Schreiner et al., 2002). Since the primary pigments conferring colorful features to these agri-food commodities are chlorophylls (green), carotenoids (yellow, orange, and red), anthocyanins (red, blue), and flavonoids (yellow), there is an intrinsic association between the color, texture, and nutritional composition of fruits and vegetables (Barrett et al., 2010). In tomato plants grown under low light intensity, the carotenoids and, in particular, lycopene biosynthesis were reduced (Dorais et al., 2001). When lettuce plants were exposed to shading conditions, the biosynthesis of anthocyanins was generally reduced in almost all the examined cultivars (Kleinhenz et al., 2003). Similarly, in red radish, plants cultivated in protected environment were compared with plants grown in an open field, and a positive correlation was reported between the development of pigment and the light intensity (Schreiner et al., 2002)

In green asparagus, the chlorophyll, ascorbic acid, and rutin (the main flavonoid with strong antioxidant properties, in asparagus, quercetin-3-*O*-beta-rutinoside), and in purple asparagus, the anthocyanin content, were shown to be positively dependent on light intensity (Guillén et al., 2008; Kohmura et al., 2008; Maeda et al., 2008, Maeda et al., 2010; Wambrauw et al., 2016). When light is under optimal level, the overall color and nutritional quality were negatively affected, the concentration of rutin decreased, and the spear color was pale (Wambrauw et al., 2016).

Moreover, radiation intensity was reported to affect GSL biosynthesis in different *Brassica* species (Ciska et al., 2000). The concentration of GSL in rape leaves (*B. rapa*) was reduced by low light, and this effect was linked to a significant decrease in the flavin-containing monooxygenases, which catalyze a key regulatory step in GSL biosynthesis (Wallsgrove and Bennett,1995). A similar low light-induced effect was reported also for broccoli and *B. oleracea* varieties (Charron et al., 2005; Schonhof et al., 2007; Cartea et al., 2011).

Finally, preharvest environmental conditions, particularly light intensity (both high and low stress), cause responses at the physiological and biochemical levels, which may impact the postharvest life of many fruits and vegetables influencing their susceptibility to postharvest disorders or causing damages directly during postharvest (Ferguson et al., 1999; Gruda, 2005). The lower level of vitamin C reported under low light environment was linked to the worst performance of fruits and vegetable to sub-optimal environmental condition during the postharvest chain (Lin and Jolliffe, 1996; Noctor and Foyer, 1998; Gruda, 2005; Witkowska and Woltering, 2010).

In the next paragraph are reported some studies on the possibility of being able to improve the quality of fruit and vegetables through moderate levels of preharvest light stresses.

## Modulation of Abiotic Stresses for Improving the Quality of Agricultural Crops

Abiotic stresses in the cropping systems negatively affect produce quality and reduce the yield of crops. Genetic improvement has been providing new cultivars with enhanced tolerance against abiotic stresses, but in certain periods of the year or in specific geographical areas, the cultivation can be carried out only under greenhouses. As described above, abiotic stresses activate the biosynthesis of bioactive compounds and mild stresses can even enhance the quality of the products. In modern and technologically equipped greenhouses, abiotic stresses can be applied at low intensity, and if opportunely managed, they can represent an innovative strategy for enhancing the product quality. The accumulation of bioactive compounds with antioxidant properties in fruit and vegetables represents an interesting research topic in terms of human nutrition and the beneficial effect of these functional molecules in the diet. The bioactive compounds are essential for plants, which can enhance the tolerance to abiotic stresses and can be also interesting as source of these molecules (**Figure 2**). Each abiotic stress can be a trigger for specific biosynthetic pathway leading to the accumulation of specific metabolites. Understanding crop responses to abiotic stresses and the mechanisms of accumulation of bioactive compounds can be used for the development of agronomic strategies for producing high-quality fruits and vegetables.

### Salinity Stress Application and Produce Quality

Hydroponic growing systems are widely used for the cultivation of fruit and vegetables. Optimized nutrient solutions can be used in these systems for improving yield and quality. During cultivation, the nutrient solution can be modified and enriched in salts.

The salinity of nutrient solution can be enriched by adding sodium chloride (NaCl) or using naturally salty water, commonly available along coastal areas in the Mediterranean regions.

Crops have specific tolerance threshold to salinity, which is usually expressed as EC of the nutrient solution or the substrate extract. The increase of salinity reduces the growth of crops, but if applied few days before harvest, it can induce positive quality variations of the products such as the reduction of nitrate accumulation in leaves. In leafy vegetables, for the commercial sales, they must have the nitrate concentration below the limits imposed by the EU regulation 1258/2011 (Cavaiuolo and Ferrante, 2014). The nitrate that is not assimilated is stored in the vacuole. Since nitrate has also an osmoregulation function, under water stress or low light intensity, it can be accumulated.

The increase of salinity in the nutrient solution reduces the nitrate accumulation (Zanin et al., 2009), because the excess of sodium is stored in the vacuole, avoiding its concentration in the cytoplasm. The high concentration of sodium in the vacuole in saline conditions avoids the accumulation of the nitrate. Moreover, the Cl^−^ is also a competitor in the uptake of nitrate ion (NO_3_^−^); therefore, the high concentration of Cl^−^ reduces the nitrate uptake at the root level and the accumulation in the tissues (Abdelgadir et al., 2005). This crop response can be exploited as a strategy for reducing the nitrate concentration in leafy vegetables such as lettuce, spinach, and rocket by increasing the salinity of nutrient solution 2–3 days before harvest.

The same strategy can be used for improving the sensory quality of tomato. It has been reported that the quality of tomato fruits with higher sodium concentration has higher sensorial quality (Restuccia et al., 2002).

The application of salty water can be only exploited in greenhouse cultivations using hydroponic systems, because the beneficial effects can only be obtained if the stress application is limited to few days before the harvest.

### Low Temperatures

In different species, low temperatures can induce anthocyanins and phenol biosynthesis. This strategy can be exploited for increasing the accumulation of these antioxidant compounds in the edible parts of different vegetables. Low temperatures are required, for example, in certain varieties of lettuce or radicchio plants for reaching the commercial color, which depends on the anthocyanins concentration.

A practical application can be exploited in protected cultivations by lowering the environmental temperature just by reducing the heating of the greenhouse few days or weeks before the harvesting. The critical step is the identification of the functional low temperatures specific for the crop of interest. Cold stress can be easily applied on crops grown in greenhouses during winter. The temperature reduction can be obtained by opening the windows and reducing the heating. This strategy is also applied for controlling the growth/height of many ornamental species. This technique is named “morning drop,” because the windows opening early in the morning induces a rapid heat loss with a drastic temperature decline. The temperature drops to about 6–7°C and can reduce or inhibit plant growth. This strategy works for different species, but it requires appropriate adjustments to avoid chilling injury and quality losses, as well.

### Wounding or Slicing

Harvesting procedures can induce wounds on the products due to cutting operations or mechanical damage derived from handling and transportation. These wounds can lead to quality losses in color alteration or tissue degradation. However, wounding induced by the slicing operations for postharvest products preparation, such as fresh-cut industries, can induce phenolic compound accumulation and enhance the produce antioxidant capacity. The crops showing highest wounding stress during harvest are baby leaf vegetables such as lettuce, spinach, and rocket. The wounds activate PAL, the key enzyme of the phenylpropanoid pathway, leading to an accumulation of secondary metabolites.

Wounding induced by slicing or chopping the fruit and leafy vegetables can be also used as postharvest strategy for improving anthocyanins or phenolic compounds concentration (Cisneros-Zevallos, 2003). Purple-flesh potatoes after slicing showed an increase of PAL enzyme activity with an increase of 60% of phenolic compounds and subsequently also an increase of the antioxidant capacity (Reyes and Cisneros-Zevallos, 2003). Analogous results have been observed in sliced lotus root (Hu et al., 2014). These evidences can be applied in the fresh-cut industries for increasing the antioxidant compounds of the products.

### High Light Intensity and UV Light Treatments

High light intensity applied as supplementary lighting in a greenhouse can induce the biosynthesis of bioactive compounds such as vitamin C and carotenoids. Ascorbic acid is an important antioxidant with well-known beneficial effects on the human health. Ascorbic acid biosynthesis comes from the primary metabolism and from fructose-6P (Akram et al., 2017). Therefore, during the fruit ripening or before the vegetables are harvested, the application of additional light intensity, such as supplemental lighting, can increase the ascorbic acid content. At the practical level, the high light treatments can be applied in the greenhouse or other indoor cultivation. It is important to balance the light intensity and environmental temperature; otherwise, the high lighting can induce an excess of light with photodamage problems. Therefore, the highest effect of light treatments can be obtained when the temperature inside the greenhouse is in the range of the optimal range for the photosynthetic activity of the crop. It means that during winter the supplemental lighting should be provided during the daylight around noon, when the temperature inside the greenhouse is the highest possible (optimal range), especially in cold countries.

High light intensity can also increase the carotenoids concentration. Carotenoids are molecules with protective function against photobleaching of chlorophylls (Biswall, 1995); hence, high light intensity can have a positive effect on leaf carotenoids concentration. The high light intensity has also a positive effect on fruit carotenoids. Tomato plants grown under high light intensity (traditional greenhouse) and low light conditions (photovoltaic greenhouse) showed different lycopene and β-carotene concentrations. The tomato fruits obtained from plants grown under high light conditions showed double the lycopene and β-carotene concentrations (Bulgari et al., 2015).

UV radiations can induce the accumulation of phenolic compounds and increase the antioxidant capacity of products. During cultivation or few days before harvesting, the UV radiation applied as pulse treatments can induce the phenylpropanoid pathway and increase the phenolic compounds.

The high light intensity can also activate the biosynthesis of phenylpropanoids (**Figure 3**). Lettuce plants exposed to high light show the accumulation of anthocyanins in leaves, which change color from green to blue. The color change is due to the activation of the phenylpropanoids pathway and in particular of the PAL enzyme and the correlated enzymes, which can lead to the accumulation of a wide range of metabolites, including anthocyanins (**Figure 3**).

The application of UV-B radiations to sweet basil with doses of 0.5, 34, 68, and 102 kJ m^−2^ day^−1^ delivered in 6 days (sub-chronic exposure) significantly enhanced the phenolic concentrations without compromising the efficiency of the PSII and the leaf functionality (Mosadegh et al., 2018).

Chili pepper (*Capsicum annuum* L.) exposed to 1.14 kJ m^−2^ day^−1^ UV-B radiation increased phenolic compounds in particular: chlorogenic acid, luteolin 8-C-hexoside, apigenin 6-C-pentoside-8-C-hexoside, and apigenin 8-C-hexoside (Rodríguez-Calzada et al., 2019). The combination of UV-B light exposure and drought can have a synergistic effect as it was observed for luteolin 6-C-pentoside-8-C-hexoside with an increase of more than 50% in chili pepper or an antagonist effect as found for apigenin 6-C-pentoside-8-C-hexoside, which showed lower levels in combined stresses (Rodríguez-Calzada et al., 2019). A practical application of UV lights can be exploited using light-emitting diode (LED) lamps, which can be set with the optimal UV spectrum (nm) and intensity for the species of interest. The highest performance and light use efficiency (LUE) can be also obtained by placing the lamps in the right positions and distance from the canopy of the crops (Cocetta et al., 2017). The LED lamps can be also placed between rows of crops such as tomato (interlighting). Since UV light can be also dangerous for the growers or operators who work in the greenhouse, it is advisable to apply the UV treatments during the night, when the energy cost is also lower.

### Moderate Water Stress Induced by DI

Water stress as described above can have a negative effect on growth and, hence, on yield and quality. However, moderate water stress applied as DI can be exploited for inducing the accumulation of bioactive compounds (Ahmed et al., 2014; Bogale et al., 2016). The most important issue is the identification of the tolerance threshold of each species and the response time after the water stress application. The crops should be in the primary response stage of the water stress. It means that the plant metabolism is partly shifted in the biosynthesis of bioactive compounds such as osmolytes, antioxidants, and plant hormones, without showing any external symptom of stress.

It is important to constantly control the soil or substrate moisture content using tensiometers or other water content sensors and contemporarily the crop stress status. The most useful non-destructive measurement of plant stress is represented by chlorophyll *a* fluorescence and derived parameters. The application of DI can be carried out using appropriate mathematical models and software that can manage the water availability in order to induce bioactive molecules biosynthesis without reducing the crop performance.

Since the aim of the DI is the accumulation of the bioactive compounds, the most important issue is to define when the water reduction must be applied and how long the treatment should be carried out before harvesting to obtain the highest bioactive compounds accumulation.

## Conclusion and Further Prospective

Abiotic stresses can be used as tools to enhance the nutraceutical quality of crops and fruits. However, the effects of practical applications can vary depending on genetic diversity, agronomical practices, environmental conditions, and the combination of all these factors. The improvement of health-related properties of products in response to abiotic stresses should be obtained without affecting the yield. For these reasons, understanding the mechanisms adopted by plants to counteract these stresses (involving both the primary and secondary metabolisms) is the key step to control the abiotic stresses and use them as tools to improve the nutraceutical properties of crops. At the same time, the individuation of physiological, biochemical, and molecular markers related to stress tolerance and linked to improved nutraceutical quality may serve in breeding and crop selection program.

The practical application of abiotic stresses for bioactive compound accumulation requires the identification of the optimal application time, the crop sensitivity threshold, the intensity of the stress to apply, and the best methods for stress control during the application.

Controlled abiotic stress can be a new frontier of the applied sciences and can lead to the production of produce with a higher nutraceutical value. At the same time, this practice can help in reducing the use of natural resources, such as water, and in enhancing usage of saline or sub-optimal cultivation environments.

## Author Contributions

The manuscript was prepared with the following contributions: AlF wrote the primary and secondary metabolism sections; GC wrote the section related to effect of salinity on nutraceutical properties; ST and DR wrote the water stress and produce quality section; RB wrote the cold stress and produce quality section; AT wrote the sub-optimal light and quality section; and AnF wrote the section related to the modulation of abiotic stresses. All authors revised and approved the final version of the manuscript.

## Funding

This work was carried out with the “Fondo per il finanziamento delle attività base di ricerca” funded by the Ministry of University and Research of Italy.

## Conflict of Interest

The authors declare that the research was conducted in the absence of any commercial or financial relationships that could be construed as a potential conflict of interest.
